# Nano electrospray gas-phase electrophoretic mobility molecular analysis (nES GEMMA) of liposomes: applicability of the technique for nano vesicle batch control

**DOI:** 10.1039/c6an00687f

**Published:** 2016-08-03

**Authors:** Victor U. Weiss, Carlos Urey, Andreas Gondikas, Monika Golesne, Gernot Friedbacher, Frank von der Kammer, Thilo Hofmann, Roland Andersson, György Marko-Varga, Martina Marchetti-Deschmann, Günter Allmaier

**Affiliations:** a Institute of Chemical Technologies and Analytics , TU Wien , Vienna , Austria . Email: guenter.allmaier@tuwien.ac.at ; Fax: +43 1 58801 16199 ; Tel: +43 1 58801 15160; b Department of Surgery , University of Lund , Lund , Sweden; c Department of Environmental Geosciences and Environmental Science Research Network , University of Vienna , Vienna , Austria; d Biomedical Center , University of Lund , Lund , Sweden

## Abstract

Liposomes are biodegradable nanoparticle vesicles consisting of a lipid bilayer encapsulating an aqueous core. Entrapped cargo material is shielded from the extra-vesicular medium and sustained release of encapsulated material can be achieved. However, application of liposomes as nano-carriers demands their characterization concerning size and size distribution, particle-number concentration, occurrence of vesicle building blocks in solution and determination of the resulting vesicle encapsulation capacity. These questions can be targeted *via* gas-phase electrophoretic mobility molecular analysis (GEMMA) based on a nano electrospray (nES) charge-reduction source. This instrument separates single-charged nanoparticles in the gas-phase according to size in a high-laminar sheath-flow by means of an orthogonal, tunable electric field. nES GEMMA analysis enables to confirm liposome integrity after passage through the instrument (in combination with atomic force microscopy) as well as to exclude vesicle aggregation. Additionally, nanoparticle diameters at peak apexes and size distribution data are obtained. Differences of hydrodynamic and dry particle diameter values, as well as the effect of number- and mass-based concentration data analysis on obtained liposome diameters are shown. Furthermore, the repeatability of liposome preparation is studied, especially upon incorporation of PEGylated lipids in the bilayer. Finally, the instruments applicability to monitor mechanical stress applied to vesicles is demonstrated.

## Abstract 2

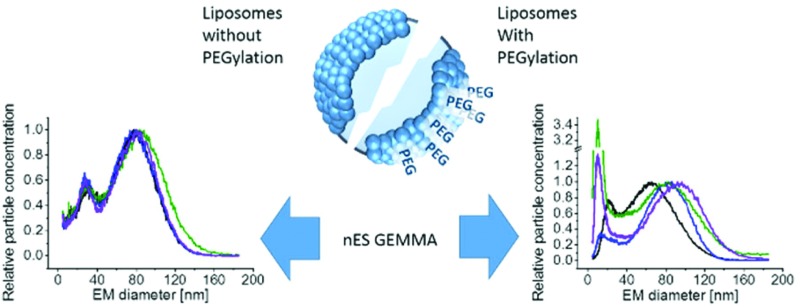
Gas-phase electrophoresis of single-charged particles enables liposome characterization and finally the resulting vesicle encapsulation capacity determination.

## Introduction

Liposomes are nanoparticles formed from a lipid bilayer encapsulating an aqueous interior. Such nano-vesicles can be classified according to their size and lamellarity.^1^ Basically, liposomes allow encapsulation (and hence protection from the aqueous extra-vesicular medium), transport and sustained release of cargo compounds when applied *e.g.* in pharmaceutical^2^ or nutraceutical applications.^3^ Cytotoxicity of drug compounds for instance often requires their shielded and targeted transport and on site release which can be achieved by application of liposomes. All these applications share their need for characterization of carrier vesicles as vesicle size in the nm range and the corresponding particle number concentration determine the drug, dye or vitamin encapsulation capacity of liposomes.

To date, various analytical setups for liposome characterization have been described. These include, imaging techniques like atomic force microscopy (AFM),^4^ transmission electron (TEM) or cryo electron microscopy (cryo EM)^5^ for visualization of particles. However, as a large number of particle images (1000 and upwards) are needed to allow for the determination of accurate particle shape and size distributions with good statistics, these methods are time consuming even with adequate software support. Furthermore, the application of high vacuum, *e.g.* in TEM or cryo EM, might lead to liposome shape distortion due to excessive interactions of analytes with sample carrier materials. Moreover, preferential enrichment of certain sample constituents of the final liposome preparation on the carrier material employed for analyte imaging has to be avoided. Nevertheless, imaging techniques yield number-concentrations of nanoparticles as recommended by the European Commission in 2011 (2011/696/EU from October 18^th^, 2011). Other analytical setups for nanoparticle characterization are liquid phase-based, *e.g.* electrophoretic separations^6,7^ or field-flow fractionations.^8^ Especially, dynamic light scattering (DLS) analysis is very popular due to its straightforward manner for size characterization of liposomes in solution.^1^ However, due to preferential detection of larger nanoparticles, information on smaller-sized sample components is often lost completely. Therefore, DLS is well-suitable to detect monodisperse nanoparticles within a sample, but is biased when nanoparticles cover a broad size range, *i.e.* are of higher polydispersity or show a multimodal size distribution.

Within this manuscript, we want to present nano electrospray gas-phase electrophoretic mobility molecular analysis (nES GEMMA) as a valuable alternative to the described established analytical methods for liposome vesicle characterization. nES GEMMA separates single-charged analytes in the gas-phase at ambient pressure according to their electrophoretic mobility diameter (EM diameter).^9,10^ In the case of spherical particles, the EM diameter corresponds to the analyte size. Single-charged analytes are obtained from a nES process with subsequent drying of nanoparticle-containing droplets and charge conditioning. The latter occurs in a bipolar atmosphere induced by a ^210^Po α-particle emitter. Depending on the size of analytes, in large part neutral analytes are obtained (which are not considered further) as well as a certain percentage of single- and, considerably less, multiple-charged particles (positive and negative, respectively).^11^ nES GEMMA results are based on data obtained from single-charged analytes. Size separation of particles occurs in the nano differential mobility analyzer (nDMA) part of the instrument in a high laminar flow of filtered (*i.e.* particle free), compressed air and an orthogonal, tunable electric field (scanning a certain voltage range). EM diameter values between 2.0 and 64.4 nm are separable at a high laminar flow value of 15 liters per minute (Lpm), *i.e.* at the maximum resolving power of the applied device. Application of lower Lpm values results in a larger EM diameter size range accessible with the used instrumental set-up. Variations in the field strength (voltage scanning) lead to deviation of charged particles from their high laminar flow imposed trajectory. Hence, only nanoparticles of a given EM diameter corresponding to an applied field strength are able to pass through the nDMA and enter the detection unit of the instrument, a condensation particle counter (CPC). In the CPC, singly-charged particles act as condensation nuclei in a supersaturated atmosphere of *n*-butanol and are subsequently counted as they pass a focused laser beam. This detection setup allows single particle and number-concentration based detection in accordance with the EC recommendation concerning nanoparticle analysis (2011/696/EU). Furthermore, the nanoparticle detection is completely independent of the chemical nature of the nanoparticles. However, it is worth mentioning that volatile electrolyte solutions for electrospraying of the nanoparticles are required as high amounts of non-volatile sample components (*e.g.* from employed buffers) are interfering with detection of the target nanoparticles.^9,12^

Several synonyms for nES GEMMA instruments can be found in the literature: macro ion mobility spectrometer (macroIMS),^13^ LiquiScan-ES (official manufacturing-company-given name of the instrument for a short time period), ES-DMA^14^ or scanning mobility particle sizer (SMPS) spectrometer.^15^ However, for matters of consistency with previous publications of others (*e.g.* ^16–19^) and our group (*e.g.* ^10,20,21^) we stick to the term nES GEMMA.

To date, several reports have been presented, targeting the analysis of liposomes with nES GEMMA-like instruments. Epstein and colleagues described in an innovative work the number-concentration measurement of liposomes in water and the comparison of their measurements to calculated values based on theoretical considerations.^22^ Their instrument focused on the detection of larger liposome particles. Hence, a so-called long DMA (lDMA) was used which allows separation of particles from approximately 15 nm up to 900 nm EM diameter at very low resolving power. Thus, in order to generate analyte containing aerosol a combination of an atomizer (different to the applied nES GEMMA system) and a diffusion dryer was employed and the integrity of liposomes was assessed finally *via* TEM. The group of Biswas on the other hand concentrated on the preparation and characterization of 100 nm liposome containing aerosol for pulmonary drug delivery.^23,24^ Either an atomizer/diffusion dryer combination was employed (comparable to the instrument of Epstein and colleagues) or a nES setup without additional drying of the nES spray gas. For the latter, 40 μm inner diameter spray capillaries were used. Liposome aggregation was observed in subsequent TEM analyses, which was suggested to be related to liposome aggregation already during the nES process. Additionally, the same group attributed an observed bimodal nano-vesicle distribution in nES GEMMA likewise to liposome aggregation during the nES process.

In addition to liposome analyses, individual samples of purified (from human blood) high density, low density and very low density lipoprotein particles (lipids as for example cholesterinester, triglycerides and protein containing macromolecule assemblies without an aqueous lumen and containing only lipid monolayers) have been size-analyzed by nES GEMMA^25^ or sera from volunteers were employed to determine lipoprotein particle sizes and concentrations in blood.^26^ Likewise, nanolipoprotein (NLP) particles,^27^ including additional membrane proteins, could be separated from bare NLPs *via* nES GEMMA.^28^

It was the aim of our work to (i) determine whether indeed single liposome vesicles are detected *via* the nES GEMMA, and not unspecific lipid aggregates or aggregates of smaller vesicles, and (ii) to answer the question of liposome integrity and aggregation after nES GEMMA passage by including subsequent AFM experiments (a non-vacuum device in contrast to TEM) after collection. Additionally, (iii) we wanted to highlight the importance of dry air application for the nES process. (iv) Moreover, ultrasonication was employed to subject vesicles to mechanical stress, which leads to liposome disruption as demonstrated from nES GEMMA measurements. Based on our data, we furthermore present (v) obtained particle size values concerning mass- and number based data evaluation. Differences between dry particle diameter values and hydrodynamic size values are shown and critically discussed. Finally, (vi) we targeted the question of liposome preparation repeatability, especially when PEGylated lipids are included in the lipid bilayer. Resulting experimental data can be employed to calculate the theoretical vesicle drug encapsulation capacity.

## Experimental

### Chemicals and reagents

Ammonium acetate (NH_4_OAc, ≥99.99%), ammonium hydroxide (ACS reagent) and Tween 20 (BioXtra) were from Sigma Aldrich (Steinheim, Germany). Chloroform (Spectronorm) was obtained from VWR (Roncello, Italy), Methanol (LiChrosolv) from Merck (Darmstadt, Germany). Atto633-COOH was purchased from Atto Tec (Siegen, Germany). Nitrogen gas was from Messer (Gumpoldskirchen, Austria). The lipids 1,2-dipalmitoyl-*sn-glycero*-3-phosphocholine (16:0 PC, DPPC), 1,2-dioctadecanoyl-*sn-glycero*-3-phosphoethanolamine (18:0 PE, DSPE), 1,2-distearoyl-*sn-glycero*-3-phosphoethanolamine-*N*-[methoxy(polyethylene glycol)-2000] (ammonium salt) (18:0 PEG2000 PE, DSPE-PEG2000) and cholesterol (Chol) were from Avanti Polar Lipids (Alabaster, AL, USA obtained *via* Instruchemie, Delfzyl, The Netherlands). Water was of Millipore grade (18.2 MΩ cm resistivity at 25 °C). NH_4_OAc (40 mmol L^–1^, pH 8.4) filtered through a 0.2 μm pore size syringe filter (surfactant free cellulose acetate membrane from Sartorius, Göttingen, Germany) was used as aqueous electrolyte.

### Liposome preparation

Liposomes were prepared according to the well-described thin lipid film hydration technique.^29^ The following lipid compositions were employed – DPPC : Chol : DSPE (6 : 3 : 1 molar ratio) and DPPC : Chol : DSPE-PEG2000 (6 : 3 : 1 molar ratio). In short, corresponding amounts of dry lipids were weighed to 50 mL round bottom glass flasks pre-cleaned with methanol : chloroform, 1 : 3 mixture (v : v), and dried (i) *via* nitrogen gas with (ii) subsequent deposition into vacuum (desiccator). Lipids were dissolved in methanol : chloroform, 1 : 3 mixture (v : v) and a thin, regular film was slowly formed under a constant stream of nitrogen gas. The lipid film was further dried in a desiccator. Hydration of the lipid film was performed with 1 mL NH_4_OAc including Atto633-COOH (10 μmol L^–1^). This yielded dispersions of 10 mmol L^–1^ total lipid concentration. Dispersions were vortexed and heated above the main phospholipids’ transition temperature (approx. 65 °C) until the lipid films had fully detached from the flask surfaces. Subsequently, dispersions were extruded for 21 times on a heating block held at a corresponding temperature to obtain small unilamellar liposomes. Extrusions were through two pre-wetted 100 nm pore size, polycarbonate membranes (Avanti Polar Lipids) applied in the same membrane orientation. Alternatively, dispersions were extruded sequentially through 400, 200 and 100 nm pore size membranes (each sequential step was carried out under conditions as for single extrusions). Liposome stock solutions were stored in glass vials at 4 °C until further use.

### Instrumentation

nES GEMMA measurements were carried out on an instrument from TSI Inc (Shoreview, MN, USA) consisting of an nES aerosol generator (Model 3480) equipped with a ^210^Po α-particle source, a nDMA (Model 3080) and a *n*-butanol-based ultrafine CPC (Model 3025A). Typical instrument settings for formation of a stable Taylor cone at the 25 μm inner diameter, fused silica capillary (TSI Inc) tip were: 4.0 pounds per square inch differential (psid, approx. 28 kPa) and 2.05 kV resulting in currents in the range of –340 nA for sample introduction to the nES capillary. 0.1 liters per minute (Lpm) CO_2_ and 1.0 Lpm compressed, particle-free air were employed for transport of the droplets through the neutralization chamber and to the nDMA unit. Particle-free air was additionally dried (Donaldson Variodry Membrane Dryer Superplus obtained *via* R. Ludvik Industriegeräte, Vienna, Austria) prior to application. Individual measurements were carried out between 5 and 184 nm EM diameter upon application of 2.5 Lpm sheath flow inside the nDMA. Four respective measurements (150 s scan time, 30 s to reset the high voltage of the nDMA to starting values, respectively, raw particle counts per detector channel were used) were combined *via* their median to yield a corresponding spectrum. The nES capillary was flushed with NH_4_OAc including Tween 20 (0.0005% [v : v]) between analyses of samples. Liposomes were collected on a silicon wafer surface for AFM imaging after passage of the nDMA *via* an electrostatic nanometer aerosol sampler (ENAS, model 3089, TSI Inc) at –3 kV and 1 Lpm air flow for 150 min.

AFM measurements were performed with a NanoScope 8 scanning probe microscope (Bruker, Santa Barbara, CA, USA) operated in tapping mode using single crystal silicon cantilevers (NCH, Bruker).

The intensity-, volume-, and number-weighted average hydrodynamic diameter of the liposome suspension was determined by DLS using a Malvern Zetasizer instrument (Malvern Instruments, Malvern, United Kingdom) using incident light (*λ* = 633 nm) scattered at 173°. Mean size was expressed as *Z*-average from cumulate fit analysis.

### Sample preparation

**Fig. 1:**
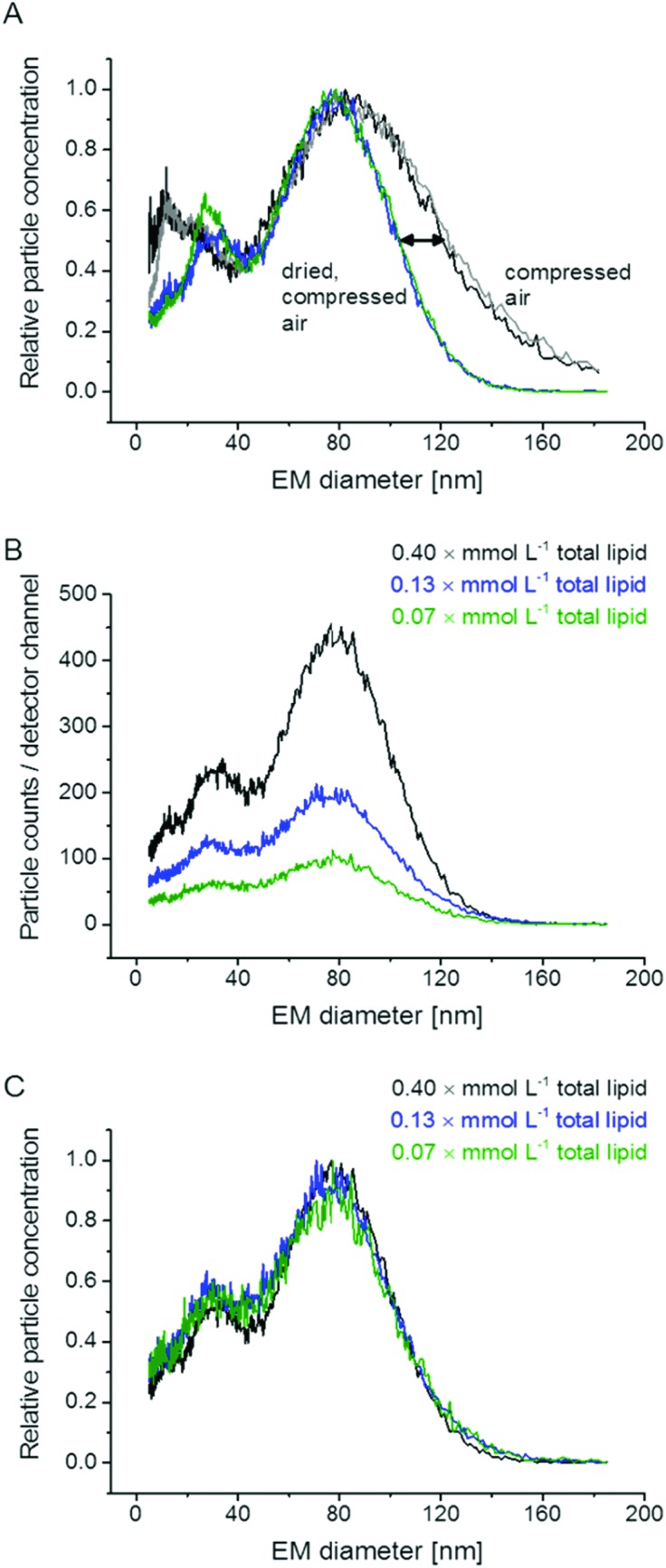
nES GEMMA measurement of liposomes; DPPC : Chol : DSPE (6 : 3 : 1 molar ratio) liposomes were subjected to nES GEMMA analysis either with or without additional drying of the compressed air employed for the nES process. In case of additional drying, solvent molecules that attach to vesicle surfaces are removed to a significantly higher extent. Hence, the recorded peak for liposome vesicles is reduced in its width (A). Unspecific aggregate formation of analytes either in solution or during the nES process is excluded as the same sample measured at different dilutions (original spectra in (B)) yields highly comparable results upon normalization (C).

Liposomes were typically diluted 1 : 25 (v : v) in NH_4_OAc to yield samples of 0.4 mmol L^–1^ total lipid concentration for nES GEMMA measurements. In case unspecific particle aggregation was investigated (Fig. 1B and C) also higher diluted samples were analyzed. Samples of 0.4 mmol L^–1^ total lipid concentration were likewise employed for AFM analysis with preceding application of an ENAS. Sonication of samples was for 30 min at ambient temperature. Samples were diluted either 1 : 100 or 1 : 1000 (v : v) in NH_4_OAc for DLS measurements without significantly different results – data for 1 : 100 (v : v) dilutions are shown.

### Data evaluation

In order to obtain EM diameter values of peak apexes as well as particle numbers, symmetrical Gauss peaks were fitted to spectra (OriginPro v 9.1.0).

## Results and discussion

As previously shown,^22–24^ GEMMA allows analysis of liposomal samples. With the current study it was our intention to better understand the influence of measurement setup parameters using nES GEMMA and to elaborate on the concept of ‘particle size’, before going into detail on liposome batch analysis and repeatability of vesicle production.

### Basic considerations concerning nES GEMMA measurements of liposome vesicles

Our initial experiments concentrated on measurement setup parameters during nES GEMMA analysis of liposomes. In doing so, we opted for a setup comprised of a nES and a nDMA allowing analysis of particles from 5 nm up to a size of 184 nm EM diameter. We decided to include a diffusion dryer in our instrumental setup to work with dry, particle free, compressed air for the nES process. As can be seen in Fig. 1A, application of dried air leads to a significant shift of the liposome peak upper EM diameter (right edge) to a lower value. It should be noted that the lower EM diameter of the peak (left edge) remained constant. We suppose that this observed shift is induced by differences in the loss of electrolytes and water molecules from the nano-vesicle's solvating envelope. Upon application of highly dried air, the amount of solvent molecules on the vesicle surface decreases, which significantly reduces liposome peak heterogeneity. The liposome peak width is reduced by roughly one third at the standard deviation *σ* of a fitted, symmetrical Gauss peak. As a direct consequence of the reduced peak width, the peak apex shifts from 85.1 to 79.9 nm EM diameter. The influence of the applied dry compressed air on the liposome vesicles themselves seems improbable, because in that case an influence on the lower liposome peak limit would have been detected. Yet, the lower peak limit remains constant for both experimental setups (compressed air, with and without additional drying). Additionally, atomic force microscopy (AFM) data supports our conclusion of intact liposome particles (see below). To conclude, similar to salt molecules attaching themselves to analyte surfaces during the nES process,^9,12^ also solvent molecules influence particle EM diameter values. Application of dried compressed air leads to improved removal of solvent molecules attached to vesicle surface and hence more homogeneous nES GEMMA peaks.

Additional experiments focused on the formation of unspecific aggregates in solution and during the nES process. As demonstrated in Fig. 1B and C, results from dilution experiments are comparable when spectra are plotted as relative data: no differences concerning peak apexes or particle size distributions are detected. Hence, no unspecific aggregates are formed in solution. Based on this data, also liposome aggregate formation during the nES process seems highly unlikely. It is to be noted that components detected with approx. 20 nm EM diameter might originate from micelles or other lipid aggregates.

**Fig. 2:**
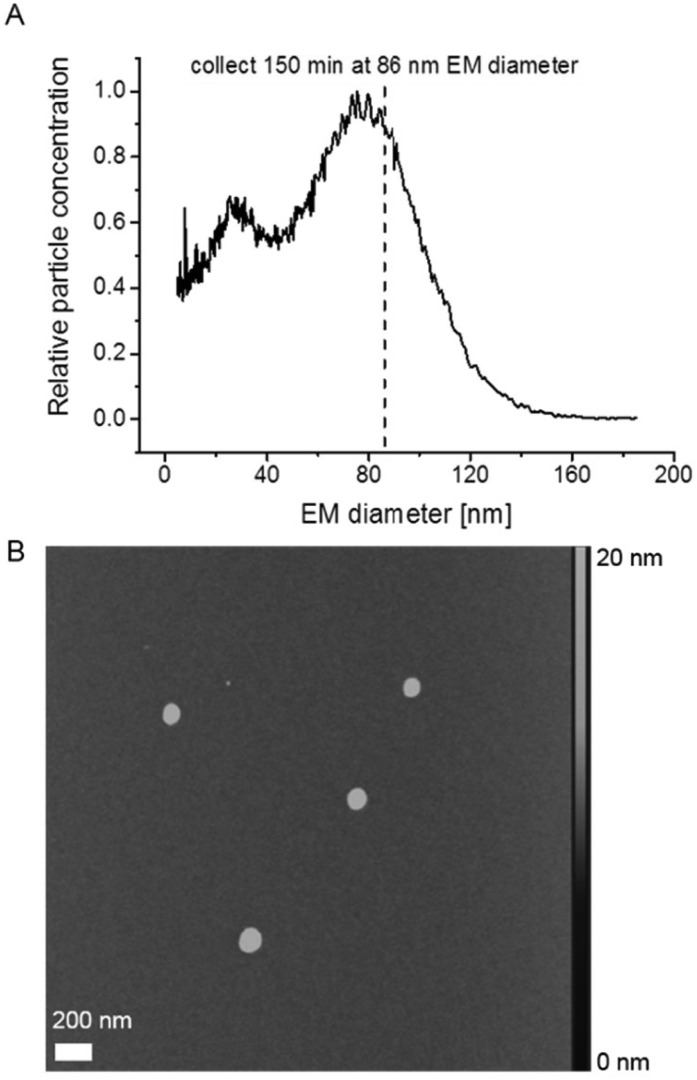
ENAS collection of liposome vesicles on a silicon wafer surface allowed for subsequent AFM investigation of particles; liposome vesicles (yielding a nES GEMMA spectrum as shown in (A)) were collected for 150 min at 86 nm EM diameter on an even silicon wafer surface. As demonstrated by AFM, collected particles are of comparable size (B).

Subsequently, we employed AFM to investigate vesicle integrity after GEMMA analysis. AFM, in contrast to TEM as previously reported,^22^ works at ambient pressure and hence we expected a reduced impact of the applied imaging method on liposome particle integrity. Fig. 2A shows a corresponding nES GEMMA spectrum of the investigated sample indicating the EM diameter at which sampling occurred. Liposomes were collected as previously described in detail^20^ for 150 min at 86 nm EM diameter on a silicon wafer surface. Initial experiments had demonstrated a sufficiently even surface of the applied silicon wafer (data not shown). Collection of liposomes *via* nES GEMMA relates spherical shaped particles exhibiting a diameter in the size range of the EM diameter at which particles were sampled (Fig. 2B). However, the height of the collected particles is significantly reduced (max. 20 nm) in comparison to their diameter (approx. 100 nm). We concluded already in a previous study^30^ that this is most likely caused by force application of the AFM tip or interaction of particles with the wafer surface, or a combination of both.

**Fig. 3:**
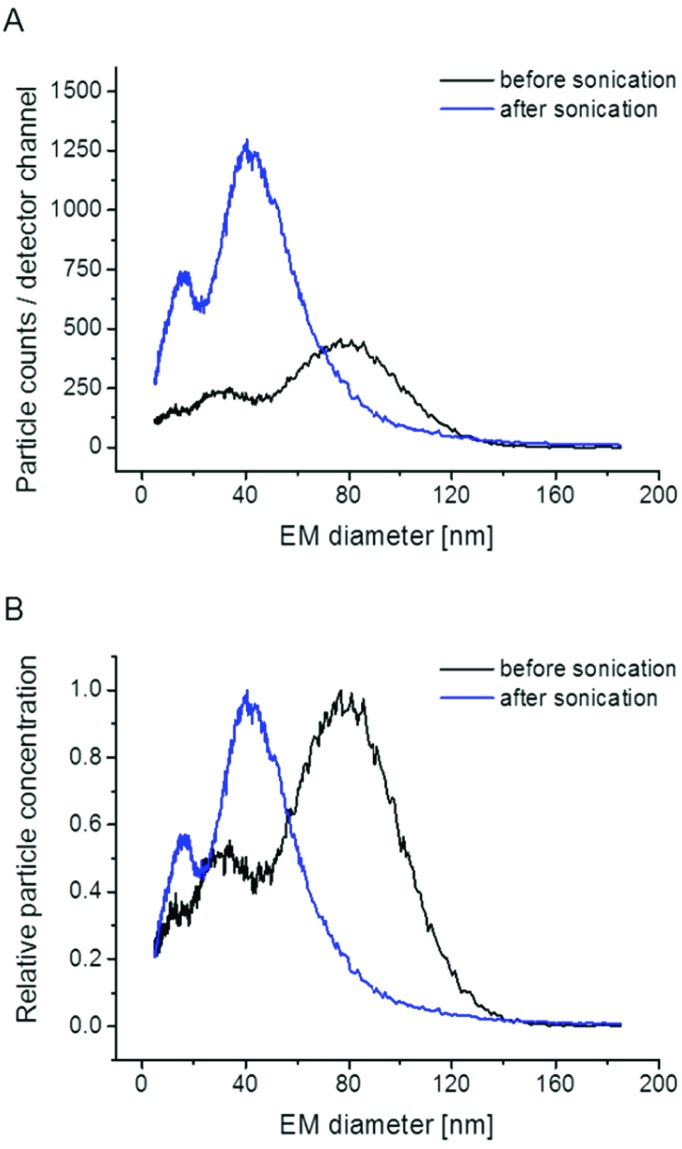
nES GEMMA spectra of liposomes prior and after sonication demonstrate the susceptibility of vesicles to mechanical stress; after sonication the peak at approx. 80 nm EM diameter peak apex was no longer detected. Instead particle populations at lower EM diameter values are increasingly found. Particle counts per detector channel (A) and relative particle concentrations (B) of the same dataset are depicted.

To further corroborate vesicle integrity during nES GEMMA measurements, sonication experiments for intended vesicle disruption are presented. As depicted in Fig. 3, sonication indeed eliminates the particle peak at approx. 80 nm EM diameter peak apex. Concomitantly, peaks at lower EM diameter values gain in their intensity as these peaks probably originate from lipid molecular aggregates, disrupted liposome bilayers or smaller vesicles. For future measurements these results indicate that nES GEMMA is an appropriate analytical technique to investigate the impact of mechanical stress on liposomes.

### What is the size of investigated liposome vesicles?

**Fig. 4:**
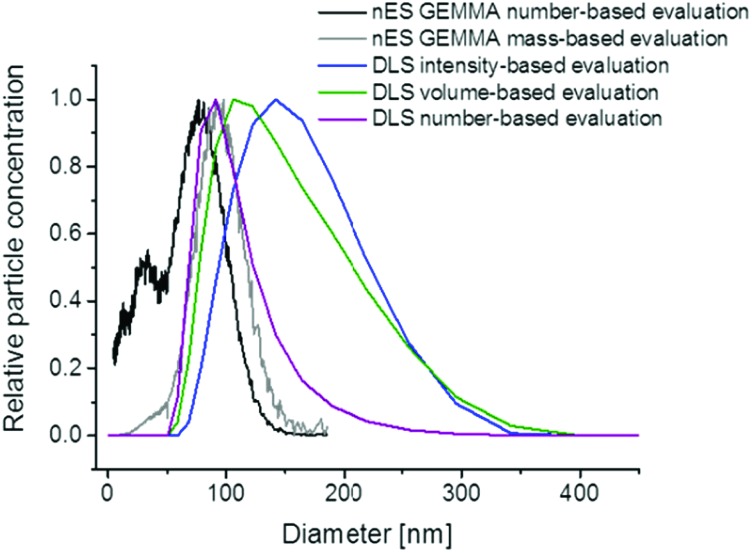
Comparison of nES GEMMA data employing number- and mass-based data evaluation with DLS data; a clear shift between dry, nES GEMMA derived particle diameters and DLS-obtained hydrodynamic particle diameters can be seen. Likewise, number- and mass-based data evaluation of nES GEMMA generated signals yields significant differences in particle diameters, as well as in detectability of low EM diameter material. This material is only accessible for analysis when nES GEMMA generated data evaluation is number-based. Mind that the identical nES GEMMA data is used for both data evaluation modes. DLS data for 1 : 100 [v : v] diluted samples is depicted.

One major characteristic of nanoparticles in general, and liposomes in the actual case, is the spatial dimension, *i.e.* the size of vesicles. However, upon vesicle size determination it is important to consider several basic principles. First of all, the mode of analyte detection (number- or mass-based) is of importance. In this context it is of note that, as shown in Fig. 4, nES GEMMA allows interpretation of originally number-based results either as number- or mass-based data (even after recording of sample spectra). In case of number-based data evaluation each nanoparticle is counted disregarding its size or mass whereas for the latter even a small number of larger nanoparticles can bias results in a way that smaller-sized nanoparticles (20 nm in the given example) are overseen, although being present in the sample in a significant number. Additionally, the apex of the liposome peak is shifted to higher values upon mass-based data evaluation (in the given example by 28%). For DLS, volume and number-based particle distributions can be calculated based on recorded light scattering intensities (Fig. 4). However, this step requires extensive knowledge of the particles optical properties and is often prone to errors. Although particle distributions are shifted to lower diameter values in doing so, still smaller sized sample components cannot be detected in the given case due to the method inherent preferential detection of larger analytes.

Secondly, it has to be considered that nES GEMMA measurements relate to dry particle diameters whereas liquid phase methods, *e.g.* DLS, obtain hydrodynamic diameter values in a corresponding solvent. Differences between these two values can be significant as was shown for the characterization of gelatin or silica based nanoparticles.^31–33^ Similar observations were recently reported for the size determination of exosomes in solution or after nanoparticle drying.^34^ Fig. 4 likewise demonstrates the significant size difference between surface-dry particles (nES GEMMA) and measurements in solution yielding the liposome's hydrodynamic diameter in NH_4_OAc. For the given analyte the DLS derived hydrodynamic diameter (based on calculated number-concentrations) exceeded measured number-based nES GEMMA data by about 23%. To conclude, the question of liposome particle size and size distribution can only be answered, if the analytical measurement technique is provided and if the physical condition in which nanoparticle size determination occurs is clearly described.^32,35^

### Characterization of liposome preparation batches *via* nES GEMMA

**Fig. 5:**
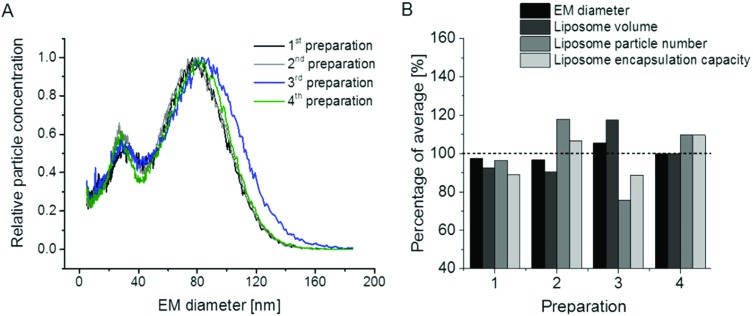
Repeatability of liposome preparation and nES GEMMA measurement of different vesicle batches; DPPC : Chol : DSPE (6 : 3 : 1 molar ratio) liposomes, extruded to 100 nm diameter (either in a single extrusion step or after serial extrusions) are compared. As demonstrated, liposome preparation and nES GEMMA spectra are highly repeatable (A). Based on EM diameter values and particle numbers, theoretical encapsulation capacities of vesicles can be assessed (B). The deviation of these values for individual batches from an average (100%, respectively) was approx. ±10%.

In the next step we concentrated on the repeatability of liposome preparations. In doing so, we prepared two additional vesicle batches comparing in total *n* = 4 liposome preparations (note that two vesicle batches – 1^st^ and 2^nd^ in Fig. 5A – were already presented in Fig. 1A). The repeatability of liposome preparation, as well as nES GEMMA measurement, is demonstrated in Fig. 5A. All four liposome batches exhibited comparable EM diameter values and similar amounts of smaller sized sample components of the bimodal size distribution. It is to be noted that for two of the liposome batches, single extrusion to 100 nm vesicle size was changed to serial extrusion steps (400 nm pore sized filters followed by 200 nm and 100 nm pore sized filters – 3^rd^ and 4^th^ preparation in Fig. 5A). Fig. 5A likewise shows that the application of single *versus* serial extrusion had no significant impact on the obtained liposome preparation. Therefore (in terms of obtained liposome carrier vesicles), application of serial extrusion steps does not seem necessary. However, when vesicles are intended for drug delivery, serial extrusion might be necessary to prevent clogging of the filter membrane due to aggregate formation of hydrophobic cargo molecules or unspecific cargo-carrier accumulations.

Subsequently, we inquired whether it is possible to deduce further parameters of liposome batches from nES GEMMA measurements besides (i) the particle size values for dry vesicles and (ii) size distributions of liposome preparations revealing the amount of undesired smaller sized sample components. Indeed, we suggest that nES GEMMA data allows (iii) to calculate the relative theoretical encapsulation capacities (EC) of vesicle preparations *via* (iv) calculation of liposomal vesicle volumes *V* = (1/6) × *π* × (EM diameter)^3^, deduced from the EM diameter of particles at the peak apex assuming spherical shape as proven by AFM, and (v) vesicle particle numbers (PN) as deduced from number concentration data, as well at the peak apex. EC is obtained as product *V* × PN. Resulting values for liposome preparations shown in Fig. 5A are given in Fig. 5B.

We are aware of the fact that the EM diameter is determined by the diameter of the lumen and the thickness of the bilayer alike, the latter being dependent on the phospholipid composition of the bilayer. However, in order to set up a generalizable and easily applicable model we applied a simplification disregarding the contribution of the bilayer to the liposome EM diameter in our approach to calculate the theoretical EC. Likewise, our simplified model neither considers multilamellarity nor the possibility of slight differences in charge conditioning between preparations as these factors were not suggested to impact the observed general trend. However, it has to be kept in mind that for a detailed model of a given vesicle batch these numbers have to be determined by orthogonal and time-consuming techniques, *e.g.* cryo EM.

Based on our generalizable as well as easily applicable model and as expected from nES GEMMA data, also the theoretical encapsulation capacity of the four liposome preparation batches was of very good repeatability. Keep in mind that the smaller-sized sample components of the bimodal size distribution (small liposomes, micelles, liposome building blocks or similar) were neglected for the calculation of the theoretical vesicle encapsulation capacity as their aqueous lumen (if any in case of micelles and unspecific aggregates) is negligible when compared to full-sized liposomes.

**Fig. 6:**
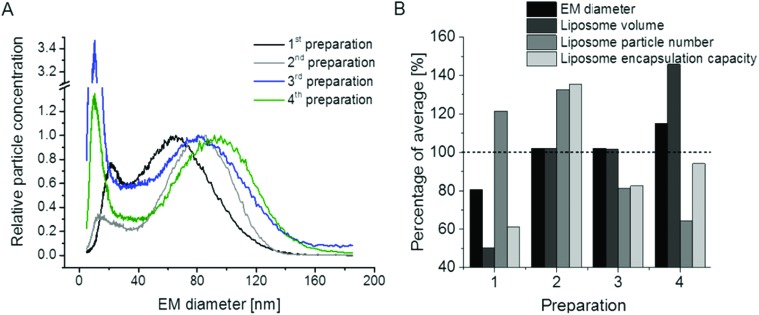
Repeatability of liposome preparation and nES GEMMA measurement of vesicle batches upon DSPE-PEG2000 incorporation in lipid bilayers; DPPC : Chol : DSPE-PEG2000 (6 : 3 : 1 molar ratio) liposomes, extruded to 100 nm diameter (either in a single extrusion step or after serial extrusions) are compared. In contrast to non-PEGylated liposomes as shown in Fig. 5, the excellent repeatability of vesicle batch preparation is lost as can be deduced from nES GEMMA spectra (A) and resulting theoretical encapsulation capacities of liposomes (B).

PEGylation of liposomes is today a recognized method to increase blood circulation (*i.e.* half-life) of vesicles during drug delivery. Therefore, in a final step, we made an attempt to perform the analysis of vesicles in which phosphoethanolamine (PE) of original liposomes was substituted with its PEGylated form, DSPE-PEG2000. Resulting GEMMA spectra of *n* = 4 preparations (likewise duplicates from single – 1^st^ and 2^nd^ – and serial extrusion experiments – 3^rd^ and 4^th^) are shown in Fig. 6A. Surprisingly, the very good repeatability of liposome preparation was lost upon application of PE in its PEGylated form. This finding is also reflected in the theoretical encapsulation capacity of vesicles (Fig. 6B), which was calculated as described above. From our experiments it appears that PEGylation greatly influences the repeatability of vesicle preparation probably due to (i) interaction of PEG side chains during lipid film and liposome formation, (ii) possibly (at least partially) disoriented PEG side chains, (iii) heterogeneity of PEG side chain length upon DSPE-PEG2000 conjugate formation and (iv) influence of PEGylation on vesicle lamellarity. Therefore, especially for liposomal carriers including PEGylated lipids the exact characterization of vesicles appears beneficial to assess the drug encapsulation capacity of carriers for which nES GEMMA seems to be a well suited analytical platform.

## Conclusion

In our study we concentrated on the applicability of nES GEMMA for the analysis of liposomes. Employment of dry compressed air during the nES process (an additional drying step *via* a diffusion dryer was included in the instrumental setup) was found beneficial for nanoparticle analysis. Dry air led to removal of solvent molecules from vesicle surfaces, which in turn led to more distinct peak shapes. Additionally, we carried out nES GEMMA measurements of a liposome sample dilution series. Results from this series together with AFM images of particles having passed the nES GEMMA setup, suggested that for vesicles of our lipid composition liposome aggregation in solution or during the nES process is highly unlikely (other than observed by the group of Biswas for liposomes of a different composition^23,24^). Instead, we attributed the occurrence of the observed bimodal analyte distribution to the existence of liposome fragments, micelles, small vesicles or lipid bilayer building blocks. This suggestion was strengthened experimentally by subjection of a liposome sample to sonication which leads to a strong shift of the liposome peak in nES GEMMA spectra. Concomitantly, peaks of lower sized material increased in intensity.

Furthermore, we closely inspected differences between two data evaluation modes of nES GEMMA spectra: number- and mass-based. Only number-based data allowed the detection of a bimodal size distribution, showing smaller and larger sized compounds within one sample. In case of mass-based data analysis, results were biased towards the detection of larger particles. Hence the latter is better suited for analyte aggregate determination. We also matched nES GEMMA to DLS data to compare EM diameter values of dry particles to hydrodynamic particle diameters. We could clearly point out that there is a significant difference between these two methods and we therefore highly recommend the combination of various independent analytical methods to allow for a most comprehensive nanoparticle characterization.

Finally, we demonstrated the repeatability of liposome preparation and nES GEMMA measurements and showed that theoretical encapsulation capacities can be calculated from particle size data and vesicle numbers in a simplified, generalized and easily applicable model. Yet we also demonstrated that vesicles carrying PEG chains exhibited a significantly lower sample preparation repeatability which was also reflected in their calculated drug encapsulation capacity scattering significantly higher around an average value than for non-PEGylated liposomes.

To summarize, we were able to demonstrate that nES GEMMA is an exceptionally well-suited method for liposome characterization. Additionally, this method allows us to assess the degree of vesicle alteration in response to mechanical stress. Likewise, batch reproducibility of vesicle preparations can be easily accessed in addition to the cargo encapsulation capacities, which can be calculated from nES GEMMA data. Especially for pharmaceutical applications, the latter is suggested to give highly valuable information on a biodegradable carrier material.

## Author contributions

Initial idea by MMD, GMV, GA and VUW; experimental design by VUW and CU; liposome preparation by CU and VUW; nES GEMMA measurements by VUW, MG and CU; DLS measurements by AG, CU and VUW; AFM measurements by GF and VUW; guidance by GA, FVDK and RA; instrumentation by GA, FVDK and TH; partly funding by VUW; all authors contributed to the manuscript.
